# A Human Immunodeficiency Virus Screening Algorithm to Address the High Rate of False-Positive Results in Pregnant Women in Japan

**DOI:** 10.1371/journal.pone.0009382

**Published:** 2010-02-23

**Authors:** Takako Shima-Sano, Rika Yamada, Kazuyo Sekita, Raleigh W. Hankins, Hiromasa Hori, Hiroshi Seto, Koji Sudo, Makiko Kondo, Kazuo Kawahara, Yuki Tsukahara, Noriyuki Inaba, Shingo Kato, Mitsunobu Imai

**Affiliations:** 1 Division of Microbiology, Kanagawa Prefectural Institute of Public Health, Kanagawa, Japan; 2 Department of Health Care Management and Planning, Graduate School of Medical and Dental Science, Tokyo Medical and Dental University, Tokyo, Japan; 3 Center for Women's and Children's Health, Department of Obstetrics and Gynecology, Ishikawa Prefectural Central Hospital, Ishikawa, Japan; 4 Health Sciences Research Institute, Incorporated, Kanagawa, Japan; 5 Hori Maternity Hospital, Kanagawa, Japan; 6 Seto Hospital, Saitama, Japan; 7 Division of Obstetrics, National Center for Child Health and Development, Tokyo, Japan; 8 Department of Obstetrics and Gynecology, Dokkyo Medical University, Tochigi, Japan; 9 Department of Microbiology and Immunology, Keio University School of Medicine, Tokyo, Japan; McGill University Health Center, Canada

## Abstract

**Background:**

Prenatal human immunodeficiency virus (HIV) testing is essential for the prevention of mother-to-child transmission. However, false-positive results of screening testing are a concern as they may cause unnecessary emotional stress to pregnant women waiting for confirmatory test results. In regions with an extremely low prevalence, the positive predictive values of screening are unacceptably low rate. Here, we propose a HIV screening algorithm consisting of serial two fourth-generation enzyme immunoassays to reduce the number of false-positive screening results.

**Methodology/Principal Findings:**

When 6461 pregnant women presenting to two maternity hospitals located in the Tokyo metropolitan area of Japan from September, 2004 to January, 2006 were tested using Enzygnost HIV Integral as a first screening test, 27 showed positive reactions. When these positive reaction samples were tested using VIDAS HIV DUO Quick as a second screening test, only one of them had a positive reaction, and the remaining 26 were nonreactive. Confirmatory Western blots and nucleic acid amplification test also showed that one was positive and the remaining 26 were negative; the subject who was positive with the confirmatory tests was identical to the subject who was positive with the second screening test. Thus, by adding the second screening test, the false-positive rate was improved from 0.4% to 0%, and the positive predictive value from 3.7% to 100%, compared with the single screening test.

**Conclusion:**

By applying our serial screening algorithm to HIV testing in maternity hospitals, many uninfected pregnant women would not need to receive confirmatory tests and be subjected to emotional turmoil while waiting for their confirmatory test results. This algorithm would be suitable for HIV testing of pregnant women living in low prevalence regions such as Japan.

## Introduction

The human immunodeficiency virus (HIV) epidemic in Japan is still at a low level compared with other developed countries, but the number of newly identified infections is increasing every year. For earlier detection and clinical and preventive services, much effort is made to implement voluntary HIV counseling and testing in a variety of health-care settings including public health centers, STD clinics, and outreach medical services. According to the report of the National AIDS Surveillance Committee, 13,894 persons with HIV/AIDS were reported between 1985 and 2007, and 1,500 new cases were reported in 2007 alone [1]. The HIV prevalence in Japan was estimated at 0.008% in 2007 [2]. Of all the HIV-infected persons reported in Japan, 71.0% were Japanese men; 11.4% were non-Japanese men; 6.2% were Japanese women; and 11.4% were non-Japanese women. Currently, about 70% of the Japanese men with HIV infection are men who have sex with men.

Although the HIV prevalence in women is very low in Japan (about 0.004%), universal HIV testing has been performed for pregnant women to prevent mother-to-child transmission since 1999 [3]. Nationwide questionnaire surveys on HIV testing in pregnant women are conducted every year. The HIV testing rate has gradually increased from 73.2% in 1999 to 97.2% in 2007. Over the 21 years between 1987 and 2007, mother-to-child transmission has occurred in only one in 219 (0.5%) HIV-infected pregnant women who received both antiretroviral therapy (ART) and a cesarean section, one in 17 (6%) women who had a cesarean section without ART, and 14 in 36 (39%) women who delivered vaginally [3].

Although prenatal HIV testing is essential for the prevention of mother-to-child transmission, there are concerns about false-positive results of screening tests [4], [5]. Positive test results may cause anxiety of HIV infection and emotional stress in pregnant women waiting for confirmatory test results. Some severe cases were covered by the mass media in 2007, leading to an official notification on the frequent observation of HIV false-positive screening results from the Ministry of Health, Labour and Welfare of Japan [6].

There has been little study on the rate of false-positive results in HIV screening testing of pregnant women in Japan. Thus, we conducted a prospective study at two maternity hospitals in the Tokyo metropolitan area to evaluate the performance of screening test, including the prevalence, false-positive rate, and positive predictive value, and proposed a new HIV screening algorithm composed of two serial tests to enable a substantial reduction in the number of false-positive results at this stage.

## Materials and Methods

### Study Setting

The study was conducted from September, 2004 to January, 2006 in two maternity hospitals located in the Tokyo metropolitan area. Each of the hospitals conducts more than 1,000 deliveries each year.

### HIV Testing

Blood samples were initially tested using Enzygnost HIV Integral (Siemens Healthcare Diagnostics, Deerfield, Illinois, USA), a fourth-generation enzyme-linked immunosorbent assay with the ability to detect HIV-1 gp41 antibody, HIV-2 gp36 antibody, and HIV-1 p24 antigen at a reference laboratory of the Health Science Research Institute Inc. (Yokohama, Japan). The Enzygnost HIV Integral can test 880 samples during each run lasting 240 min. The samples that tested positive in the initial screening were subjected to a secondary screening test and confirmatory tests, which were conducted at the Kanagawa Prefectural Institute of Public Health. The second screening test was performed using VIDAS HIV DUO Quick (bioMérieux, Marcy l'Etoile, France), a fourth-generation enzyme-linked fluorescent assay with the ability to detect HIV-1 gp160 antibody, HIV-2 gp36 antibody, and HIV-1 p24 antigen. The VIDAS HIV DUO Quick can test 60 samples during each run lasting 80 min. Confirmatory tests were performed using Western blot tests (Lab blot 1 and Lab blot 2; Bio-Rad Laboratories, Hercules, California, USA), and a nucleic acid amplification test (NAT), Amplicor HIV-1 Monitor test version 1.5 (Roche Molecular Systems, Branchburg, New Jersey, USA). HIV typing was performed using SERODIA•HIV-1/2 PA (Fujirebio, Tokyo, Japan). All the tests were conducted and interpreted as recommended by the manufacturers.

### Samples for Evaluating the Sensitivity of the Screening Tests

Ten HIV-1 seroconversion panels (PRB 936, 937, 938, 939(E), 945, 951, 952, 953, 954, and 955) and two samples from HIV p24 Antigen Mixed Titer Performance panels (PRA 201-05 and 201-17) were obtained from SeraCare Life Sciences (formerly Boston Biomedica, West Bridgewater, Massachusetts, USA). Seroconversion panels were used to evaluate the sensitivity in the early phase of infection. Three samples (PRB 936-04, PRA 201-05, and PRA 201-17) were diluted twofold serially with HIV negative human pooled plasma and were used to evaluate the antigen detection sensitivity of the enzyme immunoassay (EIA).

### Screening Algorithm

**Figure 1:**
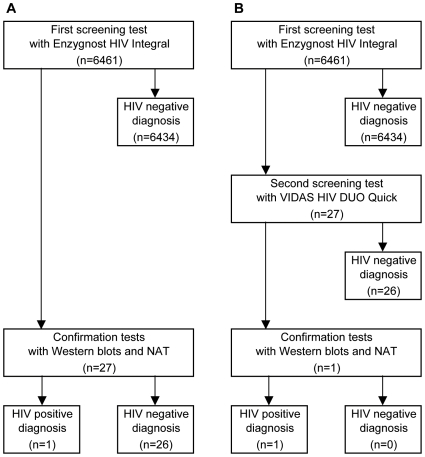
Comparison of the results obtained by two HIV testing algorithms. **A**, algorithm containing single test screening. **B**, algorithm containing serial two-test screening.

We proposed an algorithm (Fig. 1) to reduce the number of false-positive screening results in prenatal HIV testing. In our algorithm, each blood sample is tested serially with two EIA tests that should be highly sensitive and have different detection formats. The first screening test was performed on the day or next day of blood sampling during the first trimester, and the second screening test was done as soon as possible after the sample in the first test was found to be positive. If the result of the first screening test is negative, the test is reported as negative; if a result of the first screening test is positive, the same sample is tested using the second screening test. If the result of the second test is negative, the test is reported as negative; if the result of the second test is positive, confirmatory tests using Western blots and NAT are conducted.

### Statistical Analysis

Specificity was calculated using a combination of Western blots and NAT as the gold standard. Confidence intervals (CIs) were estimated using approximation to the normal distribution.

### Research Ethics

This study was jointly approved by the ethics committees at the two maternity hospitals and the Kanagawa Prefectural Institute of Public Health. The verbal informed consent for study participation including screening and confirmatory tests was obtained from study participants and recorded by the physician on a separate study-participation sheet. As blood samples used in this study had been collected as routine tests and thus no additional invasive action was required for participants, the committees approved this procedure according to the Ethical Guideline of the Ministry of Health, Labour and Welfare of Japan. All links between the test results and personal identifiers were removed and were known only to the physicians in charge of the subjects.

## Results

### Comparative Assays

The sensitivities of the Enzygnost HIV Integral and VIDAS HIV DUO Quick in the early phase of infection were compared using 10 HIV-1 seroconversion panels. HIV infection was detected with the VIDAS HIV DUO Quick earlier than with the Enzygnost HIV Integral in eight out of ten panels; the interval was an average of 4.5 days (Table 1). Next, the antigen detection sensitivities of the two tests were compared using serial twofold dilutions of three HIV-1 antigen samples (PRB936-04, PRA 201-05, and PRA201-17). The VIDAS HIV DUO Quick was 16–32 times more sensitive than the Enzygnost HIV Integral (Table 2).

**Table 1:** Test performance to detect bleed day with first positive result using seroconversion panel members.

Panel	Bleed day with first positive result		Difference in bleed days between the two tests
	Enzygnost HIV integral	VIDAS HIV DUO Quick	
PRB936	12	12	0
PRB937	21	14	7
PRB938	3	0	3
PRB939(E)	21	16	5
PRB945	13	13	0
PRB951	11	8	3
PRB952	17	10	7
PRB953	10	3	7
PRB954	21	17	4
PRB955	12	3	9
Average			4.5

**Table 2:** Antigen detection limits by antigen-antibody combined detection tests using 3 antigen positive specimens in the panels.

Panel No.	Antigen-antibody combined detection test		1∶1	1∶2	1∶4	1∶8	1∶16	1∶32	1∶64	1∶128
PRB936-04	VIDAS HIV DUO Quick	TV^1^	9.84	5.55	3.01	1.53	0.89	0.47	0.32	0.21
		Result	POS	POS	POS	POS	POS	POS	POS	NEG
	Enzygnost HIV Integral	S/CO^2^	4.52	2.32	0.90	0.64	0.38	0.21	NT^3^	NT
		Result	POS	POS	IND	NEG	NEG	NEG	NT	NT
PRA201-05	VIDAS HIV DUO Quick	TV	3.27	1.69	0.98	0.56	0.31	0.23	0.15	0.13
		Result	POS	POS	POS	POS	POS	NEG	NEG	NEG
	Enzygnost HIV Integral	S/CO	1.58	0.63	0.34	0.19	NT	NT	NT	NT
		Result	POS	NEG	NEG	NEG	NT	NT	NT	NT
PRA201-17	VIDAS HIV DUO Quick	TV	2.98	1.58	0.84	0.45	0.28	0.22	0.17	NT
		Result	POS	POS	POS	POS	POS	NEG	NEG	NT
	Enzygnost HIV Integral	S/CO	1.77	0.78	0.36	0.19	NT	NT	NT	NT
		Result	POS	NEG	NEG	NEG	NT	NT	NT	NT

POS, positive; NEG, negative; IND, indeterminate.

1 TV, test value. TV<0.25 was judged as negative, and TV≧0.25 was judged as positive.

2 S/CO, signal-to-cutoff ratio.

3 NT, not tested.

### Results of HIV Testing

Of the 6,461 study participants, 27 (0.42%) showed positive results for the first screening test performed using the Enzygnost HIV Integral. When the positive samples were tested with the second screening test performed using the VIDAS HIV DUO Quick, only one sample exhibited a positive reaction and the remaining 26 samples were nonreactive. When the samples that tested positive in the first screening were tested using confirmatory Western blots and NAT, only one sample was positive and the other 26 samples were negative (Fig. 1). The subject whose confirmatory test results were positive was the same as the one whose second screening test result was positive. The sample from this subject was positive with an HIV-1 Western blot, indeterminate with an HIV-2 Western blot, and HIV-1 positive on HIV typing. As for the signal-to-cutoff (S/CO) ratio on the Enzygnost HIV Integral used as the first screening test, the one positive sample was 6.47; of 26 false-positive samples, two were ≥6.0, four were 2.0–6.0, and 20 were <2.0. Western blots of the 26 negative samples showed that one was indeterminate with both HIV-1 and HIV-2 Western blots (S/CO ratio, 1.24), two were indeterminate with only HIV-1 Western blots (0.92 and 5.87), and one was indeterminate with only HIV-2 Western blots (6.34): all were negative on HIV typing.

In the standard protocol using a single screening test, the false-positive rate was 0.40% (95% CI, 0.25–0.56%), and the positive predictive value was 3.7%. However, when an additional screening test was introduced, the overall specificity of the screening was improved dramatically, and the above values were changed to 0% and 100%, respectively.

## Discussion

According to the guideline for prevention of mother-to-child transmission of HIV in Japan [7], women who are found to be pregnant at hospital are generally tested for HIV during the first trimester, and HIV-positive women are treated with antiretroviral therapy from the second trimester and intravenous administration of zidovudine during labor. Babies are treated with zidovudine syrup for 6 weeks after birth. Testing of women in labor is performed with a rapid antibody test, and positive women are regarded as infected with HIV, and zidovudine administration was initiated to the woman and a newborn baby.

Routine HIV testing for pregnant women has been underway since 1999 in Japan. A questionnaire survey conducted in 2003 reported that only 7 out of 82,290 pregnant women were diagnosed as being HIV-1 positive [8]; thus, the prevalence was 0.009%, which is extremely low compared with 0.15%–5% in the United States [9]. This survey also reported that the false-positive rate of screening tests was 0.094%, and that its positive predictive value was 8.3%. These values agree with those obtained in the present study. Since about one million pregnant women are tested each year in Japan, about 1,000 women are probably notified of false-positive results after screening tests.

Identifying such false-positive results using confirmatory testing is not easy. With the introduction of fourth-generation EIA tests for screening, the confirmatory test sequence has become very complicated. Because these EIA tests can detect antibodies against HIV-1 and HIV-2 as well as HIV-1 antigen, the confirmation of positive results requires an HIV-1 Western blot, HIV-2 Western blot, and NAT for HIV-1 RNA. Western blots result in a high percentage of indeterminate results [10]. Furthermore, even if the result of HIV-2 Western blot is negative, HIV-2 infection cannot be denied because the sensitivity of HIV-2 Western blots is lower than those of EIA tests for the detection of HIV-2 antibody. Therefore, even if samples are shown to be negative with any one of the three tests, the subjects should be retested one month later. Consequently, once a pregnant woman is assigned a positive screening test result by a false-positive reaction, she must undergo two rounds of confirmatory tests in one month.

HIV-tested pregnant women have been reported to encounter various problems associated with false-positive screening results [3], [9]–[13]. In Japan, pregnant women who were notified of positive screening results felt strong anxiety and depression while waiting for the results of the confirmatory tests; some of the women became suspicious of their partners, and some considered abortion or divorce [3]. One woman was notified of an HIV positive result by her clinician without receiving sufficient explanation about the screening testing; later, when confirmatory testing showed that she was HIV negative, she became upset and untrusting of medical services [3]. Such emotional disturbances have been reported in other countries [9], [10]. However, the situation in Japan may be somewhat different from those in most of developed countries because the HIV prevalence (0.009%) is extremely low. Most obstetricians have never treated an HIV-infected individual and thus have little chance of learning HIV infection and its diagnosis. Therefore, it is the most important to help obstetricians to understand the nature of HIV testing and to provide clients with counseling and information, including the high frequency of false-positive results in screening testing and the necessity of confirmatory testing to obtain a decisive result.

As an alternative approach to resolving these problems from a technical point of view, we proposed a screening algorithm consisting of serial two fourth-generation enzyme immunoassays to reduce the number of false-positive test results. When this algorithm was applied to the 6,461 pregnant women who participated in this study, the specificity of screening was improved from 99.6% to 100% and the positive predictive value was improved from 3.7% to 100%, compared with the standard protocol. Although the two screening tests were conducted at separate reference laboratories in this clinical trial, these tests can be sequentially done at the same place. By applying this algorithm to clinical settings, many uninfected clients would not need to receive confirmatory tests and thus would not be subjected to emotional turmoil while waiting for their confirmatory test results. In addition, because extensive confirmatory tests and repeat visits are not required, it results in cost savings. Although the number of participants in this study is limited, the increase in specificity and positive predictive value can likely be extrapolated to a larger population of pregnant women.

It has been suggested that false positives may be caused by alloantibodies resulting from pregnancy, transfusions, or transplantation [10]. We did not collect other medical conditions of the study participants, which may influence testing results. The false-positive rate of Enzygnost HIV Integral in this study was 0.40%, which is within a range (0.3%–0.8%) of previously reported false-positive rate of this kit [14]–[16]. Therefore, it is unlikely that the false-positive rate observed in the first screening was influenced by medical conditions including pregnancy and testing factors such as quality control and performance in the reference laboratory.

The proposed protocol is characterized by a specificity-optimized serial two-test algorithm. When the specificity is optimized, a serial testing algorithm and a parallel testing algorithm are equally sensitive and specific. A parallel testing algorithm is time-saving for diagnosis of HIV infection; a serial testing algorithm is cost-effective and less laborious. Because the HIV prevalence was very low in Japan, most of positive results are due to false positives of the tests. Furthermore, simultaneous two screening tests are not covered the public medical insurance. We think that a serial testing is more acceptable in our country.

The order of the two tests could be determined based on several factors including throughput, cost, labor intensity, sensitivity, and specificity. In this study, the VIDAS HIV DUO Quick was used as the second screening test because it is less suitable for large-scale testing and has been shown to be more sensitive in the early phase of infection than the Enzygnost HIV Integral. The latter characteristic may help to reduce the number of false-negative results in the second screening. However, it should be noted that the overall sensitivity and specificity of a serial screening algorithm are determined only by the combination of the two tests and their order is irrelevant.

Inevitably, the sensitivity of the serial screening algorithm is lower than those of the individual tests employed therein, and the specificity is higher. However, if the two tests employed are highly sensitive, the decrease in sensitivity is expected to be marginal and less than the difference among currently available screening tests [17]. On the other hand, the adoption of the algorithm improves the specificity dramatically. Theoretically, the sensitivity of the first test should be as high as possible to ensure the detection of the largest possible number of HIV-positive samples [17], [18]. However, recent EIA tests have undergone dramatic improvements, and most fourth-generation tests have achieved nearly 100% sensitivity on HIV-1/2 reference positive samples and are capable of detecting early infection two to seven days after NAT [15], [19], [20]. The ranking of these tests on seroconversion panels varies among individual panels [15], [19], [20], and is probably indecisive for field samples. Therefore, as long as two highly sensitive fourth-generation tests are used, which test is more sensitive may not be the primary determinant of the test order.

We should be cautious in applying the proposed screening algorithm to other health-care settings. In clinical settings specializing in HIV infection, clinicians are likely to diagnose their patients with a high sensitivity and specificity. The HIV-1 prevalence in this setting would likely be relatively high, and the clinicians would be well trained with regard to providing counseling and information regarding HIV testing. In such a setting, the present algorithm may not be required. Meanwhile, voluntary HIV counseling and testing have been implemented chiefly in public health centers on a free-of-charge basis, and rapid antibody tests are widely used for screening testing. The sensitivity and specificity of third-generation rapid test were shown to be lower than those of fourth-generation EIA tests [16], [17]. In these cases, the introduction of a fourth-generation EIA test as the second screening would not miss true positive samples and would enable a great reduction in false-positive screening samples.

As the performance of HIV diagnostic tools evolve, the diagnostic algorithms should also be changed to be accurate as well as beneficial to the clients, and they need to be developed specifically for individual health-care settings. The screening algorithm presented in this study provides improved specificity and positive predictive value, and cost savings, which is suitable and beneficial for HIV testing in low prevalence settings such as for maternity hospitals in Japan.
